# Editorial Perspective: On the need for clarity about attachment terminology

**DOI:** 10.1111/jcpp.13675

**Published:** 2022-08-02

**Authors:** Marije L. Verhage, Anne Tharner, Robbie Duschinsky, Guy Bosmans, R. M. Pasco Fearon

**Affiliations:** ^1^ Clinical Child and Family Studies Vrije Universiteit Amsterdam Amsterdam The Netherlands; ^2^ Amsterdam Public Health Research Institute Amsterdam The Netherlands; ^3^ Department of Public Health and Primary Care University of Cambridge Cambridge UK; ^4^ Clinical Psychology KU Leuven Leuven Belgium; ^5^ Centre for Family Research, Department of Psychology University of Cambridge Cambridge UK

**Keywords:** Attachment, terminology, language, dialogue, confusion

## Abstract

Part of the appeal of attachment language is that it feels near to our everyday experience, as terms like ‘attachment’, ‘security’ or ‘disorganisation’ feel readily recognisable. Yet, not one of these terms is used by academic attachment researchers in line with ordinary language. This has hindered the evidence‐based use of attachment in practice, the feedback loop from practice to research and the dialogue between attachment researchers in developmental psychology and in social psychology. This paper pinpoints the difficulties arising from the existence of multiple versions of ‘attachment theory’ that use exactly the same terms, held by communities that assume that they are referring to the same thing and with little infrastructure to help them discover otherwise. When we talk past one another, the different communities with a stake in knowledge of attachment are obstructed from genuinely learning from one another, drawing on their respective strengths and pursuing collaborations. One factor contributing to this situation has been the use of attachment terminology with technical meanings, but often without setting out clear definitions. We here introduce a guide to attachment terminology used by the academic community, which has recently been published on the website of the Society for Emotion and Attachm ent Studies. The guide is meant for researchers, clinicians and everyone concerned with attachment to increase understanding of the technical meaning of important terminology used by researchers, and support the quality of discussions between researchers, and between researchers and clinicians and other publics.

Part of the appeal of attachment language is that it is astonishingly evocative and feels near to our ordinary experience. Terms like ‘attachment’, ‘security’, ‘sensitivity’, ‘disorganisation’, ‘coherence’ and ‘anxiety’ feel readily recognisable and pack an emotional punch. Yet, not one of these terms is used by academic attachment researchers in line with ordinary language. This has hindered the evidence‐based use of attachment in practice. Clinicians incorporating attachment theory in their therapeutic practices may get tripped up or misled by the different meanings attributed to attachment‐related words by researchers compared to their ordinary connotations. This article describes the risks of researchers and clinicians talking past one another and the benefits of clarity about how the research community has been using attachment terminology. It also introduces a guide to this terminology, which may be used as a reference and to improve the dialogue between researchers and clinicians and others interested in attachment.

A recent study on the beliefs of researchers and clinicians on attachment theory, research findings and use in clinical practice showed three distinct belief patterns: one in researchers and two in clinicians (Beckwith, 2021). The belief patterns of clinicians depended on their backgrounds: clinicians with a background in developmental psychology held more empirical and theoretical beliefs, whereas the belief patterns of clinicians from a wider variety of therapeutic backgrounds with much clinical experience had more emphasis on practice wisdom and personal knowledge. Striking differences between the belief patterns of researchers and clinicians were beliefs about the effects of early attachment on brain development (clinicians) and sensitivity as the most effective target of intervention (researchers). Furthermore, the beliefs that were most distinctive between the two groups of clinicians (developmentalists and autodidactic therapists) included beliefs that the use of attachment language is more helpful for clinical practice compared to the use of specific attachment measures and attachment classifications (developmentalists) and the belief that attachment insecurity in itself is problematic for children (autodidactic therapists). This study clarifies that there are differences in assumptions about attachment and its implications between researchers and clinicians and among clinicians, who nonetheless may use the same terminology to refer to these different accounts.

Beckwith found remarkable differences between researchers and clinicians regarding the meaning of disorganised attachment. In her sample, one of the distinguishing features of clinicians, in contrast to researchers, was belief that disorganised attachment is always linked to maltreatment and childhood trauma. Following the ordinary language associations of the word ‘disorganised’, it is often regarded as a ‘pathological form of attachment’ or ‘attachment disorder’ (see also Granqvist et al., 2017 for a more detailed description). However, such assumptions are contrary to available scientific evidence. There is a threat here, since with such views on disorganised attachment, clinicians more likely take a blaming stance to parents or feel more pressured to take measures to protect the child identified as disorganised attached. This could hinder the development of constructive cooperation between parents and children, and increase the risk of inappropriate child removal (Bosmans, Bakermans‐Kranenburg, Vervliet, Verhees, & van IJzendoorn, 2020). In turn, researchers have not been able to benefit from clinical insight and wisdom in understanding children's displays of conflict, confusion or apprehension towards their caregivers, since misapprehension about the meaning of disorganised attachment has derailed the conversation. Greater clarity about attachment terminology is a critical basis for successful dialogue between researchers and applied professionals, where they can understand and learn from one another.

In a sense this ‘confusion of tongues’ between ordinary language and technical meanings is a longstanding and standard problem for psychology as a discipline (e.g. Derksen, 1997). Psychology attempts to characterise and support change within the sphere of ordinary life, and therefore begins with the terms and problems of ordinary language. In doing so, it develops technical meanings. To give an example: The use of the word ‘depression’ in our ordinary speech diverges from the clinical use of the term – and both probably, in turn, differ from how the term is used by academic researchers. In some regions of psychology, the problem is particularly acute, for example in psychoanalysis. For instance, the ordinary language and technical meanings of key psychoanalytic terms, like ‘sexuality’, have minimal overlap.

In attachment research confusion about the meaning of terminology has become a relatively acute problem, though perhaps not as acute as in psychoanalysis. A reason for this is that Bowlby developed the originating language of attachment theory with two goals in mind (as described in Duschinsky, 2020). On the one hand, he wanted to appeal to a popular audience, and so needed his claims to resonate with everyday concerns. On the other hand, he also wanted to use the same language to do technical work in developing scientific‐deductive hypotheses. In addition, Bowlby used terms inconsistently at times or with a lack of clarity, especially in his early – and most widely read – works. For example, whereas attachment theory implies qualitative differences in attachment (secure vs. insecure), a common misconception is that some home‐reared children are ‘more’ or ‘less’ attached to their caregiver than other children, and this misconception was encouraged by some of Bowlby's early statements.

Over the time, there has been the emergence of multiple versions of ‘attachment theory’ that use exactly the same terms, held by communities that assume that they are referring to the same thing and with little infrastructure to help them discover otherwise. This situation has been hard to rectify. Attachment research has had comparatively strong platforms for reporting and synthesising empirical findings but weak platforms for clarification of concepts and terminology, and weak platforms for supporting clinicians and other interested stakeholders to engage with the technical aspects of methods and concepts. A case in point is Ainsworth's sensitivity scale, containing her ground‐breaking operationalisation of a central attachment concept, which circulated only in manuscript form for decades among researchers, and was published only in 2015 (Ainsworth, Blehar, Waters, & Wall, 2015). It is hardly surprising that this technical concept has been widely misunderstood, with audiences assuming that the concept meant what the term ‘sensitivity’ would connote in ordinary language. In turn, what clinicians learn about sensitivity has been obstructed from feeding back into attachment research, since there has not been an effective commonly understood language for sustaining this dialogue.

Aligned observations have also been made by Waters and McIntosh (2011, p. 474):

In psychology, and more so, attachment theory, the words we use to label ideas often get in the way. They misdirect us in what we think we should do next. Many implications that people draw from their knowledge of attachment theory are probably not rigorously derived from the logic of the underlying theory. Take this example: you ask a college class, what kinds of developmental problems might arise from being insecure in your attachment to your mother? They start thinking that insecure sounds like afraid, fearful, anxious, shy, uncomfortable, maybe incompetent, and the reasoning goes on to a conclusion that insecure is therefore a bad thing. This is not being deduced from some mechanism that is spelled out in attachment theory. It is merely associative.

A further source of difficulty stems from the fact that attachment theory is currently used both in developmental psychology and in social psychology. The latter research domain introduced novel terminology like anxious and avoidant attachment styles, but also a different research approach. Developmental research focuses more on observed behaviour and individual differences in how individuals talk about attachment relationships and experiences. Social psychology focuses more on self‐reported attachment‐related expectations. The two research traditions have often used comparable terminology, such as ‘avoidance’, to refer to quite distinct psychological processes. To further complicate things, child psychiatry research introduced the concept of attachment disorder (e.g. Zeanah & Gleason, 2010). While the discussion on whether or not attachment and attachment disorders are linked is still on‐going, it added new attachment‐related language that complicated the bridge between research and clinical practice (Allen, 2016). This has further made academic attachment language yet more difficult to interpret for clinicians and other audiences, who will not be familiar with these research tradition‐related nuances.

In other regions of psychology, the problem is less acute. So for instance Cognitive Behavioural Therapy (CBT) has had relatively better coherence in the use of concepts between researchers and clinicians, since there is relatively more effective infrastructure for monitoring, evaluating and refining the use of concepts through dialogue and collaboration between clinicians and researchers (Bosmans, 2016). CBT's big strength has always been that it started from very clearly defined constructs that could be rigorously studied, often with experimental research designs allowing to manipulate these constructs and hypothesised mechanisms. As a result, both CBT researchers and therapists have a common understanding of core constructs like cognitive schemas, cognitions, information processing biases, rumination, fear or avoidance behaviour. Those clearly defined constructs allowed more rigorous and experimental research moving the theory on behaviour and cognitive processes significantly forward. Striking examples are learning studies showing activation and reactivation patterns relevant to understand repair and relapse (Craske, Hermans, & Vervliet, 2018) and cognitive bias modification research showing that information processing biases have a causal influence on the stability and malleability of expectations (MacLeod & Mathews, 2012). Finally, this research leads to a better understanding of the requirements for treatment to become more successful, which helps to increase treatment effects (Craske et al., 2018).

There are good reasons to believe that the same level of concreteness could be achieved for attachment theory, and that fundamental research and applied practice would benefit. One step towards this will be to reduce confusion about attachment terminology. For this reason, Duschinsky et al. (2021) attempted to sketch and typify diverging uses of terms such as ‘attachment’, ‘security’, ‘internal working model’, ‘disorganisation’ in developmental psychology, social psychology, psychotherapy, psychiatry, social work and popular discourse (for the example of ‘Security’, see Table 1). They have also considered the respective goals of these different constituencies, and why they may have incentives to use attachment terms in particular and diverging ways – and not to acknowledge this. All of these groups have their strengths and insights; their different perspectives should be valued. However at the same time, we perceive that risk of miscommunication would be reduced and opportunities for meaningful collaboration supported if there were greater clarity about the technical meanings given to attachment terminology by researchers.

**Table 1:** Typification of differences in conceptualisations of ‘security’

‘Security’	
Popular discourses	A good and confident psychological state, and is presented as a desired state for everyone.
Developmental science	The perceived availability of a safe haven in one's attachment figure(s) (“felt security”).
Social psychological science	The absence of attachment anxiety and avoidance.
Psychotherapy	The mechanism of good mental health in the therapeutic relationship, and in a client's other interactions.
Child welfare practice	A good parent–child relationship, indexing a child's best interest.

From: Duschinsky et al. (2021). Six attachment discourses: convergence, divergence and relay. Attachment & Human Development.

## Towards clarity regarding the use of attachment terminology

To clarify the technical meanings of attachment terminology as used in scientific research, a guide including common terms used by the academic community has recently been published on the website of the Society for Emotion and Attachment Studies. This guide is a table based on the book ‘Cornerstones of Attachment Research’ (Duschinsky, 2020) and the article ‘Six attachment discourses’ (Duschinsky et al., 2021), which draw from an extensive historical study of attachment research and its uptake by knowledge stakeholders. The table presents key concepts of attachment theory. The table first describes common misunderstandings about the concept, identified by the research of Duschinsky and colleagues. It then outlines how attachment researchers currently understand these concepts, building on the most recent evidence base. The guide is meant as a reference for researchers, clinicians and everyone working in the field of attachment, to help improve the quality of our dialogue with one another. We set out to offer an overview of the most frequently used terms related to attachment, with brief and usable outlines of the technical meanings of the attachment theoretical terminology. An excerpt of the guide can be found in Table 2. The guide is intended as a living document, under the aegis of the Society for Emotion and Attachment Studies, one that we hope that various stakeholders in attachment knowledge will feel the right to discuss, debate and revisit. We envision the current version of this guide as a starting point, to be further elaborated and adapted over time. With contributions of researchers, clinicians and others who use attachment knowledge in their work, we hope this guide will contribute to establishing a more universal use of attachment terminology to improve the dialogue among clinicians and to facilitate the feedback loop between practice and academia.

**Table 2:** Excerpt of the guide to attachment theoretical concepts

Concepts	Common misconceptions	Definition/explanation
Attachment behaviour	Instinctive behaviour of children towards their parents; clinging to the parent	Any behaviour can be an attachment behaviour when it is directed towards gaining and maintaining the availability of an attachment figure when the attachment system is active. It is not an ‘instinctual’ pre‐set pattern of behaviour. The expression of attachment behaviour can vary between situations and developmental stages. In infancy, common attachment behaviours include smiling, crawling towards the caregiver, reaching and clinging, and directed cries to attract the caregiver's attention.
Attachment classification/pattern	Four boxes representing different ‘kinds’ of attachment; ‘good’ or ‘bad’ attachment; ‘more’ or ‘less’ attached	Categories for describing individual differences in patterns of attachment behaviour in a specific relationship based on valid and reliable assessment tools.
Children		The most commonly used tool for children between 12 and 24 months is the Strange Situation Procedure (SSP). The four classifications based on the SSP are: secure, avoidant, resistant/ambivalent, disorganised. All categories that are not secure can be considered as insecure.
Adults		For adults, the most commonly used (observational, i.e. interview) tool is the Adult Attachment Interview (AAI). The four classifications based on the AAI are: autonomous, dismissing, preoccupied, unresolved (for loss and/or abuse).
Attachment relationship	A state characterising the whole of a child's relationship with his or her mother; parent–child bonding; a characteristic of the child or of the parent	A relationship between a child and another person, who functions as an attachment figure for the child. As long as a caregiver is sufficiently familiar and the relationship sufficiently stable over time, children will develop an attachment relationship with this caregiver. An attachment relationship may exist even if the attachment figure is rejecting or abusive. The quality of the care provided does not determine whether or not an attachment relationship develops, but rather shapes whether the attachment relationship is secure or insecure. This also means that a child can form multiple attachment relationships with different attachment figures. The security of these attachment relationships is relationship specific (and cannot be transferred).
Disorganised attachment	Attachment disorder; pathology of the child; the consequence of child maltreatment; chaotic, random or inexplicable behaviour of the child in any given situation; extends to unresolved/disorganised attachment state of mind in adulthood; weak attachment or lack of attachment	An attachment classification coded on the basis of relationship‐specific behaviour that appears disoriented, conflicted or apprehensive with regards to a caregiver when the attachment system is activated (e.g. in the Strange Situation). It is inferred from such behaviour that there has been some degree of systematic disruption in the functioning of the attachment system, in that the child is not able to coherently direct their attention to their caregiver (as in security or resistance) or to the environment (as in avoidance).
Secure attachment	‘Good’ attachment; characteristic of the child; leads to healthy development; leads to autonomous attachment state of mind	Attachment classification which is a relationship‐specific behavioural pattern characterised by the capacity to readily use the caregiver as a safe haven when alarmed and to accept and make use of comfort, and to use the caregiver as a secure base when the attachment system is not activated. In the infant Strange Situation it is coded on the basis of relatively high levels of proximity seeking (and often also contact maintaining behaviour) towards the caregiver when the attachment system is activated (e.g. in the Strange Situation), combined with relatively low avoidance and resistance towards the caregiver. In older children, direct proximity‐seeking becomes relatively less important for security, except when a child is scared; under more ordinary stressful circumstances, of greater importance in revealing security are expectations about the caregiver's availability as a safe haven and secure base revealed through child–parent communication or the child's stories or play.
Security	Personal wellbeing and confidence; good relationship/bonding with parents, ‘felt security’	A characteristic of the caregiver‐child attachment relationship, the confidence children have in their ability to use their caregivers as a source of comfort when alarmed, and a base from which to explore when calm. For secure attachment as the category label for a group of infants in the Strange Situation Procedure, see ‘Secure attachment’.
Sensitivity	Warmth and tenderness; personality characteristic, sensibility	As defined by Ainsworth, the ability of a caregiver to (a) perceive and to (b) interpret accurately the signals and communications implicit in an infant's behaviour, and given this understanding, to (c) respond to them appropriately and (d) promptly. Ainsworth developed a scale for assessing caregiver sensitivity. Various other measures of sensitivity have subsequently been developed by attachment researchers. Not all of them measure sensitivity as technically defined by Ainsworth.

From: Society for Emotion and Attachment Studies (2021).
